# The Cause, Prevention, and Cure of So-Called Frost-Bite

**Published:** 1918-02

**Authors:** 


***The Cause, Prevention, and Cure of So-Called Frost-Bite.*** By Victor Raymond and Jacques Parisot. Trans. and Abs. from the *Comptes Rendus de l'Académie des Sciences,* May, 1916, p. 693.

The authors state that in their study of the causes responsible for trench feet, they gradually eliminated all of those commonly mentioned, such as cold, inactivity, and compression, finding that only cold humidity was the invariable causal condition. It occurred to them that it might in some way favor an infection.

They observed that not only men with cases of trench feet, but also others who had remained for a considerable time in the trenches, developed mycoses about the nails, especially of the big toe, which is the part of the foot earliest and most severely affected by the malady.

Culture tests of the local lesions tended to support the mycosis theory. A fairly rich flora was obtained. Besides inocuous organisms like the Penicillium glaucum, a fungus of a brownish gray color, was obtained in pure culture. It was identified by Vuillemin as the Scopulariopsis Koningii Oudemans. It was first described in 1912 by Jannin. It appears to originate in dung and straw. It has been isolated in a large number of trench feet cases, especially from the blisters and scabs. The liquid of the edema and of the blisters, however, appears slightly or not at all fertile.

When the Scopulariopsis Koningii was inoculated in animals, lesions very similar to those observed in human cases of trench feet were produced. In some cases ulcers were produced causing death. Alterations of the viscera were sometimes observed.

In some cases another organism was isolated either alone or together with the Scopulariopsis. This has been identified as a Sterigmatocystis.

The authors conclude that the ordinary frost-bite of the trenches is in reality mycosis of the feet, similar to the Madura foot.

The organism is commonly found in the soil, and thrives especially in mud and stagnant water at a temperature of from 25° to 30° C.

*Treatment.* The authors recommend thorough cleansing of the feet with soap and camphorated borate solutions. By way of prophylaxis they urge the wearing of waterproof stockings or shoes, and the disinfection of the feet by camphorated and borated soaps.

